# The effects of Pilates training on abdominal muscle thickness and core endurance in patients with Parkinson’s disease: a single-blind controlled clinical study

**DOI:** 10.55730/1300-0144.5663

**Published:** 2023-04-04

**Authors:** Evrim GÖZ, Seher ÖZYÜREK, Burçin AKTAR, Berril DÖNMEZ ÇOLAKOĞLU, Birgül BALCI

**Affiliations:** 1 Department of Physiotherapy and Rehabilitation, Faculty of Health Sciences, Tarsus University, Mersin, Turkiye; 2 Department of Musculoskeletal Physiotherapy, Faculty of Physical Therapy and Rehabilitation, Dokuz Eylül University, İzmir, Turkiye; 3 Institute of Health Science, Dokuz Eylül University, İzmir, Turkiye; 4 Department of Neurology, Faculty of Medicine, Dokuz Eylül University, İzmir, Turkiye; 5 Department of Neurologic Physiotherapy, Faculty of Physical Therapy and Rehabilitation, Dokuz Eylül University, İzmir, Turkiye

**Keywords:** Parkinson’s disease, Pilates, muscle thickness, core endurance

## Abstract

**Background/aim:**

This study aims at examining the effects of Pilates training on the transversus abdominis (TrA) and internal obliques (IO) muscle thickness and core endurance in different positions in patients with Parkinson’s Disease.

**Materials and methods:**

Patients were divided into 2 groups as Pilates training (n = 13) and control (n = 10) groups. Pilates training was performed twice a week for 6 weeks. The participants’ muscle thickness and core endurance were measured at the beginning of the study (pretraining), and the effectiveness of Pilates training was then assessed in the 6th week (posttraining) and in the 12th-week follow-up. The thickness of the TrA and IO muscles was measured with a two-dimensional ultrasonography device. Core endurance was assessed with prone bridge and sit-ups tests.

**Results:**

The assessments of the Pilates training group after the 6-week showed a statistically significant increase in the prone bridge and sit-ups test performances, and an increase in the thickness of the IO muscle [during resting in the supine position and abdominal drawing-in maneuver (ADIM) in standing position] and the TrA muscle (during ADIM in the standing position) (p < 0.05). Pilates exercises were also shown to have improved core muscle endurance and thickness (IO thickness during ADIM in the standing position and TrA thickness during resting in the supine position, ADIM in the supine and standing position) in the Pilates training group after a period of 18 weeks (p < 0.05).

**Conclusion:**

Pilates training has a favorable effect on the core endurance, and the TrA and IO muscle thickness of patients with Parkinson’s disease and this effect can be maintained until three months after the training.

## 1. Introduction

Parkinson’s disease (PD) is a progressive and neurodegenerative disease with both motor and nonmotor symptoms [1]. Despite optimal medical treatment methods, patients with PD experience deterioration of body functions, and a decrease in their daily activities and overall quality of life (QoL). Therefore, usually, a therapeutic exercise approach is followed to supplement the pharmacological and surgical treatments in order to increase the functional abilities of patients with PD and to reduce the likelihood of any secondary complications they may develop [2,3]. As a result, the use of Pilates as a therapeutic exercise method is becoming increasingly popular. Pilates training has proven to be an effective method to increase flexibility, muscle strength, endurance, balance, and postural control in healthy individuals [4]. It has also been shown to improve postural stability, range of motion, mobility, and QoL in PD while mitigating the symptoms of the disease [5,7].

Pilates training focuses on the activation of core muscles such as the transversus abdominis (TrA), multifidus, rectus abdominis, external obliques (EO), and internal obliques (IO). These local and global muscles work in synergy to stabilize the core. The local stabilization system includes deeper muscles such as the IO and TrA that play an important role in increasing segmental control and the stability of the lumbar spine [8,9]. The six principles of Pilates (breathing, concentration, centering, control, precision, and rhythm) should be performed correctly in the exercise program to enhance core stabilization [10]. Pilates training increases the performance of the TrA and IO when performing daily life activities and provides a more neutral stabilization of the spine in positions that increase the load on the trunk [11].

Head and trunk control, which are supported by core muscles in terms of strength and endurance, are critical to maintaining dynamic stability during locomotion. Optimal core strength is required to properly perform the movements coordinated between the trunk and extremities during daily activities [12,13]. In previous ultrasonographic imaging (USG) studies, it was reported that trunk muscle mass was lower in elderly individuals compared to younger ones. In PD, it is believed that muscle degeneration occurs as a result of the disease symptoms as well as aging, and accordingly, segmental control is affected by the deterioration of the activation of trunk muscles, and dynamic postural control may be impaired [12–15]. In a study examining the relationship between the thickness of the trunk muscles such as erector spinae and multifidus and the sagittal spinal alignment in PD, it was reported that there is a correlation between the decrease in the thickness of the trunk muscles and the decrease in the lumbar lordosis. In addition, it was emphasized that it is important to seek effective strength training methods to improve the thickness of the trunk muscles in patients with PD [16].

Pilates training also increases the strength and endurance of the abdominal muscles as well as the thickness of the core muscles [17]. Rehabilitative ultrasound imaging (RUSI), a real-time, reliable, fast, and cost-effective method has been frequently used in the literature to measure the changes in the thickness of the trunk (including the core) muscles [18–20]. However, since abdominal drawing-in maneuver (ADIM) is a fundamental component of core exercises that improve core stability, it is frequently used together with RUSI to measure the activation of the deep abdominal muscles [11,21]. Additionally, ADIM promotes selective contraction of the transversus abdominis while maintaining minimum contraction of internal oblique and external oblique muscles [22].

Previous studies in the literature have shown that the thickness and activity of the core muscles change during different positions and functions. Studies have stated that the TrA and IO muscle thicknesses are higher in the standing position than in the supine position. A possible explanation for this is that the base of support is reduced in the standing position compared to the supine position, and more activation of the TrA and IO muscles is needed to provide spinal stability in this challenging position [23,24]. In view of such information, we decided that it is necessary to assess TrA and IO muscle thicknesses in different positions (supine and standing) and during different functions such as resting and ADIM.

There are sample studies which reported that Pilates training increased TrA and IO thickness in studies conducted with healthy subjects; however, there are no studies conducted with PD patients. In addition, in these studies, measurements are usually taken in the supine position, and measurements taken in the standing upright posture, which is a more challenging posture in terms of activation of the core muscles, are limited. Therefore, we aimed to investigate the effects of Pilates training on core endurance and TrA and IO muscle thickness in different positions in patients with PD.

## 2. Materials and methods

### 2.1. Participants

The study was designed as a single-blind controlled study and the data were collected from May 2018 to March 2020. This research has been registered to ClinicalTrials. ClinicalTrials ID is NCT04619784. It was approved by the Ethics Committee of Dokuz Eylül University (protocol number: 3958-GOA, approval number: 2018/11-06, approval date: 03.05.2018) and performed in accordance with the Declaration of Helsinki principles. All subjects willing to participate signed written informed consent.

The sample size calculation was based on a similar study which showed a 1.9-mm increase in IO thickness after Pilates training [25,26]. The minimum sample size was calculated to be at least 20 people to meet the 95% confidence interval and 80% power in the OpenEpi version 3.0 (www.OpenEpi.com) program.

#### Inclusion criteria

- Diagnosis of idiopathic PD,
- Mini-mental test ≥24,
- Modified Hoehn and Yahr stage ≤3,
- Age ≥18 years,
- A stable dopaminergic drug regimen.

#### Exclusion criteria

- Neurological, orthopedic or visual dysfunctions other than PD,
- History of surgery related to PD,
- Participation in a physiotherapy treatment in the last 6 months,
- Refusal to participate in the study.

Patients who could not tolerate Pilates training or wanted to leave the study voluntarily were excluded.

### 2.2. Procedure

Patients were divided into the Pilates training group (PTG) and the control group (CG). The patients were evaluated during the “ON” stage. Assessments were performed in both groups by investigators blind to the treatment at the baseline (pretraining), in the 6th week (posttraining) and in the 12th-week follow-up (12 weeks after the end of the intervention). The treatment was done by a physiotherapist who is a certified Pilates trainer and has 7 years of experience in neurological physiotherapy (E.G.). Ultrasound imaging was done by a physiotherapist who is certified in ultrasonographic (USG) imaging and has 12 years of experience in musculoskeletal physiotherapy (S.Ö.). No side effects or complications were observed in the participants of this study.

**Figure 1:**
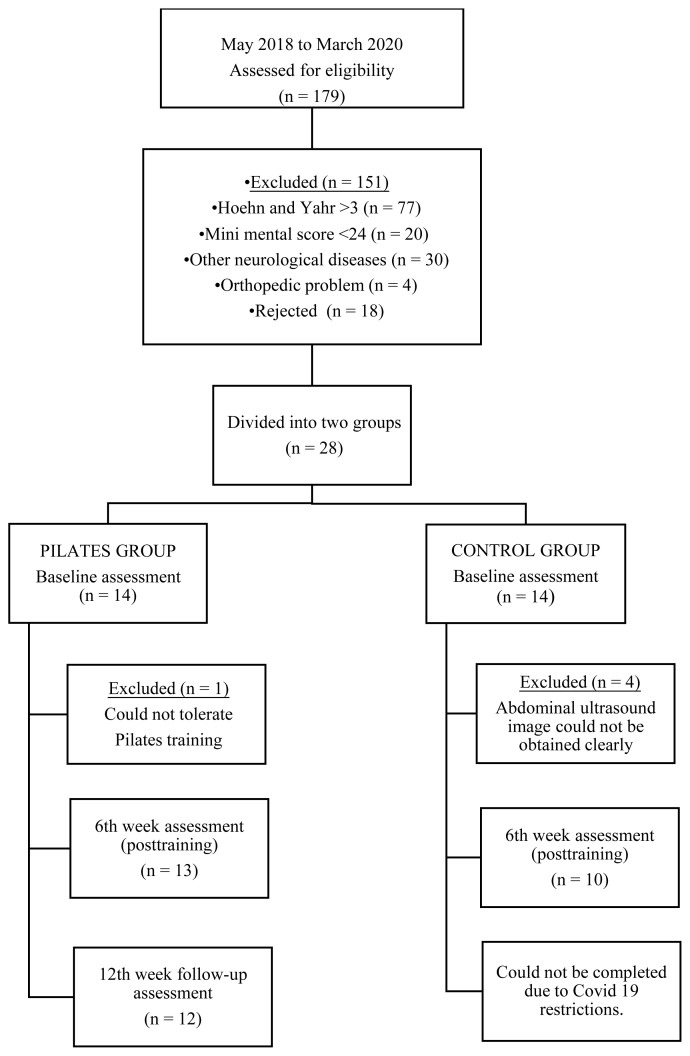
Flowchart of study process.

The study started with 28 patients (PTG = 14, CG = 14). The posttraining evaluation was completed with 23 patients due to exclusions. One patient in the PTG did not attend the 18th-week evaluation and therefore the study was completed with a total of 22 patients (PTG = 12, CG = 10). The flowchart of the study is presented in Figure 1.

### 2.3. Interventions

During the research period, all patients continued their routine dopaminergic treatments at the same dose.

#### 2.3.1. Pilates training group (PTG)

After the baseline evaluations, the patients were given an average of 1 h of Pilates training in a group of 2 or 3 people, 2 days a week for 6 weeks. The number of exercise repetitions started at 10–12 and increased to 20 as of the 4th week. Before the training, an instruction session was held to inform the patients about Pilates principles and exercises. Patients were informed about the basic principles including breathing, concentration, centering, control, precision, and rhythm, and they were instructed to focus on these principles and maintain them during exercise. Each exercise was demonstrated in practice by the physiotherapist first. The physiotherapist controlled the exercises throughout the training and used some cues for necessary corrections. Progress in the difficulty level of Pilates training was achieved by changing the body positions during the exercise, exercise ball, and band use according to the performance of the patients and based on Pilates principles after 3 weeks [27]. At the end of the supervised training program, the patients were asked to continue doing the same exercises at home for another 12 weeks. The training program is shown in Table 1.

#### 2.3.2. Control group (CG)

During the research period, all patients continued their routine dopaminergic treatments at the same dose in the CG. In this group, the baseline and 6th-week evaluations were completed, but the 12th-week follow-up evaluations could not be performed because the majority of the patients could not attend due to Covid-19 restrictions.

### 2.4. Measurements

Evaluations were made at baseline, in the 6th week, and in the 12th-week follow-up (12 weeks after the end of the intervention) in the PTG and CG.

#### 2.4.1. Clinical assessments

##### Modified Hoehn and Yahr (mH&Y)

Clinical features of the patients were evaluated with mH&Y, which defines 5 main and 2 intermediate stages of disease progression. Stage 1 shows the lowest disease severity and stage 5 shows the highest disease severity [28].

##### Movement disorder society-unified Parkinson’s disease rating scale (MDS-UPDRS)

The scale, which consists of four parts, was published by the MDS in 2009 and the Turkish version of the scale was standardized. Nonmotor problems and treatment complications are evaluated in the first part, daily activities of the patients in the second part, motor findings in the third part, and motor complications in the fourth part [29,30]. Sections 1, 2, and 3 of the scale were adopted in this study.

mH&Y and MDS-UPDRS scores of all patients were evaluated by the same neurologist (B.D.Ç.).

#### 2.4.2. Abdominal muscle thickness

**Figure 2:**
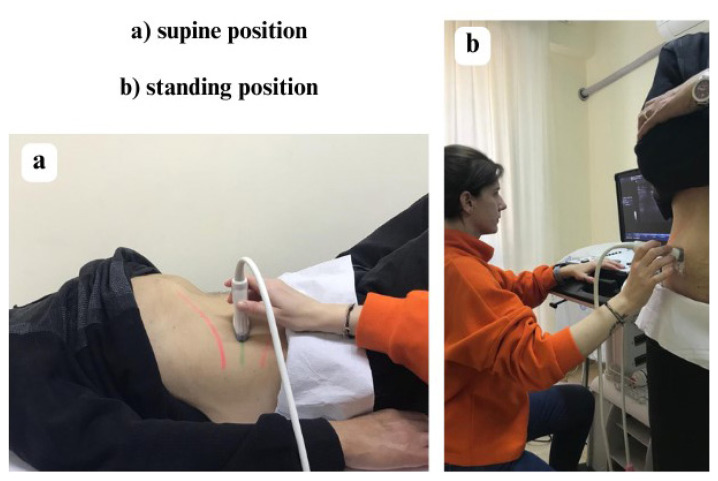
Ultrasound images of right anterolateral abdominal muscles at rest (a) and during abdominal drawing-in maneuver (b). IO, internal oblique; TrA, transversus abdominis.

**Figure 3:**
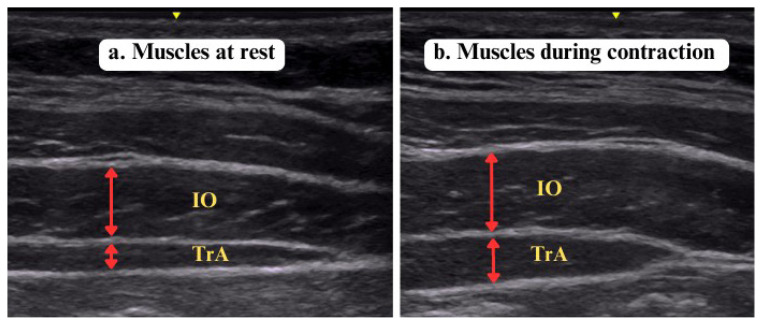
Ultrasound images of right anterolateral abdominal muscles at rest (a) and during abdominal drawing-in maneuver (b). IO, internal oblique; TrA, transversus abdominis.

Ultrasound imaging was performed to measure the TrA and IO muscle thickness by using a two-dimensional ultrasonography device (LOGIQ-e, GE Healthcare, Milwaukee, WI, USA). The systematic reviews indicated that RUSI is a valid measurement with a good correlation with MRI and EMG in measuring the size and activation of trunk muscles during isometric submaximal contractions, including TrA and IO muscles, they also established that RUSI has intra- and intermeasurement reliability [18–20]. Ultrasound imaging was done in both supine (lying with knees flexed at 45 degrees–Figure 2a) and standing positions (Figure 2b). All images were collected and processed by a single investigator (S.Ö.) with six years of experience in musculoskeletal USG imaging (accredited by The Royal College of Radiologists, the British Medical Ultrasound Society, and the European Society of Musculoskeletal Radiology). A multifrequency linear array transducer (GE 12L-RS, bandwidth 5–13 MHz, footprint 12.7 × 47.1 mm) at a central frequency of 10 MHz was used for capturing B-mode images. The images were taken by placing a probe on the right abdominal wall anterolaterally at the level of the axillary line, vertically at the midpoint of the distance between the inferior angle of the rib cage and the iliac crest. Measurements were repeated at the end of calm expiration (at rest, Figure 3a) and abdominal drawing-in maneuver (ADIM) activity (Figure 3b). Before the test, the physiotherapist taught all the patients how to do ADIM, which involves pulling the navel upwards and inwards without any excessive movement in the superficial abdominal muscles. During the test, the patients were asked to comfortably inhale and exhale, to pull in without moving their spine after exhaling, and measurements were made during the maneuver [11,31–33]. The muscle thickness measurements (in cm) were made between the superficial and deep hyperechoic fascial lines. Three measurements were taken and their average was recorded for statistical analysis.

#### 2.4.3. Core muscles endurance

##### Prone bridge test

Static endurance of the core muscles was assessed with the prone bridge test. In this test, patients were asked to lift their trunks by placing their weight on the forearms and toes while their elbows were in the flexed position. Measurements were recorded in seconds using a stopwatch, and the test was terminated when the test position was disturbed. Each measurement was made twice, and the best measurement was used in the statistical analysis [34].

##### Sit-ups test

The dynamic endurance of the core muscles was assessed with the sit-ups test. In this test, patients were asked to flex the trunk with the knees flexed and the feet stabilized. The number of repetitions in 30 s was recorded [34].

### 2.5. Statistical analysis

All data were analyzed using IBM SPSS (version 22.0) software. The normality of distribution was examined using visual (histogram and probability graphs) and analytical methods (Kolmogorov-Smirnov/Shapiro-Wilk test). The comparison of the demographic, clinical, and muscle measurements between the two groups was done with the Mann-Whitney U test. The statistical significance level was found to be p < 0.05. The Friedman test was used to examine the effects of Pilates training on core muscles’ endurance and thickness (three assessments: pretraining, posttraining, and 12th-week follow-up). The results were then analyzed by posthoc tests (Wilcoxon signed ranks test with Bonferroni correction) and the significance level was set at 0.017 after Bonferroni correction.

## 3. Results

A total of 23 patients’ data were analyzed. The demographic and clinical characteristics of the patients are shown in Table 2. The results show no statistically significant difference between the groups in terms of sex, age, height, body weight, disease duration, and modified Hoehn & Yahr scale and MDS-UPDRS scores. The core muscle endurance and muscle thickness were found to be similar in both groups, except for TrA during resting in the standing position (p > 0.05, Table 2).

Table 3 presents the comparison of the differences observed in muscle endurance and muscle thickness between assessments (pretraining and posttraining) in two patient groups.

The results showed the endurance of the core muscles (according to the prone bridge and sit-ups tests) in the PTG to be higher than in the CG after 6 weeks of Pilates training they got. The endurance of the core muscles improved in the PTG after the exercise program, while it worsened in the CG (p = 0.005, p = 0.002, respectively, Table 3).

It was observed that the muscle thickness of IO during resting in the supine position and during ADIM in the standing position increased in the PTG and decreased in the CG (p = 0.008, p = 0.005 respectively). In addition, the muscle thickness of TrA during ADIM in the standing position was seen to have improved with Pilates exercises in the PTG (p = 0.003, Table 3).

There was also a statistically significant increase in the endurance of core muscles according to pretraining, posttraining, and follow-up measurements in the PTG (p < 0.05, Table 4). In this group, there were significant increases in the TrA muscle thickness during resting in the supine position, during ADIM in the supine position, and during ADIM in the standing position at the pretraining, posttraining, and follow-up assessments. Similarly, a significant improvement was observed in IO muscle thickness during ADIM in the standing position at the end of the 18-week period including the pretraining, posttraining, and follow-up assessments (p < 0.05, Table 4).

## 4. Discussion

The aim of this study was to investigate the effects of Pilates training on core endurance and TrA and IO muscle thickness of patients with PD in different positions (such as standing and supine). In this study, core endurance, which was evaluated with prone bridge and sit-ups tests, was found to have improved in the PTG and decreased in the CG. Similarly, TrA muscle thickness during ADIM in the standing position, and IO muscle thickness during ADIM in the standing position, and during resting in the supine position increased in the PTG and decreased in the CG.

In recent studies, evaluations with magnetic resonance imaging and ultrasound after Pilates training have shown that hypertrophy is achieved in the abdominal wall muscles. In addition, it has been reported that an improvement in TrA muscle thickness is possible with the correct ADIM training during Pilates exercises in healthy subjects [11,35]. In sedentary healthy women, it was shown that the thickness of the abdominal wall muscles increased after 8 weeks of Pilates training (EO, IO, and TrA muscles during rest) [25]. In our study, when the pretraining, posttraining, and follow-up results were examined in the PTG, it was seen that TrA muscle thickness increased during resting and ADIM in the supine position and during ADIM in the standing position. This increase in TrA muscle thickness is consistent with the results in the literature [11,25].

Pilates is frequently used in the rehabilitation field as it activates the TrA and IO muscles for spinal stabilization. Han et al. investigated the effects of 6 weeks of Pilates training on trunk muscle thickness (TrA, IO, EO, multifidus, rectus abdominis) and balance (Romberg and Stability limits test) in healthy individuals. Their study indicated that IO, EO, and multifidus muscle thickness increased more and balance improved more in the PTG than in the CG [26]. Similarly, in our study, it was observed that IO muscle thickness during resting in the supine position, IO and TrA muscle thickness at the standing position during ADIM increased in the PTG, while it decreased in the CG. In our study, since two groups consisted of patients with PD, the reduction of muscle thickness in the control subjects was considered normal due to the progressive nature of the disease which causes the deterioration of muscles. In another study, TrA muscle thickness measured during the hundreds of exercises was seen to have increased significantly in the PTG, but the IO muscle thickness evaluated in the ADIM position was observed to have decreased after 8 weeks of Pilates training. However, no change was observed in the muscle thickness in the CG that did conventional strengthening exercises. It was also stated that there was no change in muscle thickness in functional positions (sitting and standing) in both groups and the thickness change was observed only during the Pilates training [11]. In our study, it was observed that while the thickness of both TrA and IO muscles decreased in the CG during ADIM in the standing position, it increased in the PTG, and this improvement was maintained for a long time in the PTG (based on the measurements done in the 12th week). Critchley et al. stated that if the patients do not get proper instruction on how to do ADIM, this may lead to the measurement of muscle thickness being erroneous or staying unchanged [11]. For this reason, we organized an introductory session before the evaluation in order to prevent this problem, and in this session, the patients were taught both the basic elements of Pilates training and ADIM. In addition, in the study of Critchley et al., the exercises were given to the patients as a home program after a single training session. However, in our study, the exercises were supervised for the whole 6 weeks by a physiotherapist, who has a Pilates training certificate. We believe that the activation of the TrA and IO muscles in the functional position is an outcome of this. The expected changes in TrA and IO muscle thickness, which were measured during functional activities after Pilates training, could not be obtained in previous studies in the literature that did not include functional positions in the Pilates program. However, in this study, when the patients were taught how to contract the core muscles in functional positions during Pilates training, it was observed that the thickness of the core muscles (especially TrA and IO) increased more [11,17]. In our study, the Pilates program was modified according to the needs of patients with PD, and it was designed to include exercises in different functional positions such as supine, prone, sitting, crawling, and standing. Therefore, we contend that the positive results of the training given in different positions contributed to an increase in muscle thickness.

Pilates has recently been used in neurological rehabilitation to increase the strength and endurance of the core muscles [36–38]. Bulguroğlu et al. compared the core endurance results of patients who did Mat Pilates and Reformer Pilates, and a control group of patients with multiple sclerosis and found significant improvements in prone bridge and sit-ups tests performances of the patients in the Pilates groups [34]. Similarly, in our study, the core endurance, which was evaluated with prone bridge and sit-ups tests, increased in the PTG and decreased in the CG. Furthermore, the improvement of the core endurance in patients was preserved for 3 months after Pilates training. We think that this change in core stability is related to the increased activity of the TrA and IO muscles. We also believe that this change is related to ADIM, which is a maneuver used continuously during Pilates exercises and especially activates the TrA muscle [22].

In the literature, it has been shown that the strength of the trunk and abdominal muscles progressively decreases in stroke patients, similar to PD, and it has been stated that the ability to control the trunk predicts the future functional ability to perform activities of daily living [39]. Lee et al. demonstrated that trunk stability training was effective in increasing TrA and IO muscle thickness in stroke patients, and accordingly, patients’ balance and walking ability also improved. Therefore, they stated that a selective trunk stability exercise program may be effective in improving daily functions and trunk control ability in stroke patients [40]. In another study investigating the relationship between the thickness and contractility of the abdominal muscles, balance disorders, and risk of falling in stroke patients, it was shown that the contractility ratio of the abdominal muscles reflects in the balance disorders and the risk of falling. However, it was also stated that resting thickness alone is not sufficient to evaluate the functioning of the abdominal muscles [41]. Also, Madokoro et al. found that TrA and IO muscle thicknesses in the supine and sitting positions were significantly greater during the ADIM than during bracing or resting. In addition, they showed that there is a correlation between the difficulty of function and muscle thickness [42]. Measuring the muscle thickness also shows the improvements in muscle after therapeutic exercise approaches in PD, it is, therefore, important to determine the changes in abdominal muscles in PD. However, to the best of our knowledge, there is no study in the literature examining the effects of stability or strengthening training on abdominal muscle thickness (during different positions and functions) in PD. This is the first study that assesses abdominal muscle thickness during different positions and functions after exercise training (Pilates) in PD.

There are studies in the literature examining the effectiveness of Pilates exercises in PD patients, but our study is unique in that it measures the strength and thickness changes in the core muscles as well as monitoring the long-term effectiveness of Pilates exercises with the follow-up measurements at 3 months after the training.

There are inevitably some limitations in this study. The small sample size could be regarded as the main limitation. In addition, nonrandomized group allocation design is another limitation. Finally, another limitation is that the follow-up assessment in the majority of the control group could not be completed because of the Covid-19 pandemic.

In conclusion, we think that Pilates exercises can improve the core stabilization of patients with PD and prevent the development of disease-specific trunk related problems in the future. We believe that since Pilates exercises are suitable for group training, it also has the potential to increase the motivation of the patients and contribute to the continuation of their participation in the exercise programs.

## Figures and Tables

**Table 1:** Pilates training.

Program		
Warm-up exercises 10 min	Breathing Centering Neck, trunk, and extremity mobility exercises	
Pilates exercises 40 min	“Shoulder drop” “Chestlift 1–2” “Hundred” “Single leg circles” “Single leg stretch”/ “Crisscross” “Side to side” “Shoulder bridge 1–2” “Book opening”	“Up/down side kicks”/”Clam” “Side lift 1–2” “Spine stretch forward” “Spine twist” “Press up” “Modified scarecrow” “Single leg kicks” “Modified swimming”
Cool-down exercises 10 min	“Child’s pose” “Modified mermaid” “Lunges” “Knee lifts” “Standing balance”	
**Total 60 min**		

**Table 2:** Comparison of demographic characteristics, disease-specific clinical characteristic, core muscle endurance and abdominal muscle thickness before the treatment.

Variable	Pilates group (n = 13) Mean ± SD	Control group (n = 10) Mean ± SD	p

Sex (n, %)			
Female	2 (15.39)	1 10.0)	0.71
Male	11 (84.61)	9 (90.0)	

Age (years)	65.62 ± 8.94	68.20 ± 7.69	0.60

Height (m)	1.72 ± 0.07	1.68 ± 0.059	0.09

Weight (kg)	80.00 ± 10.58	74.10 ± 9.90	0.23

BMI (kg/m^2^)	26.86 ± 2.60	26.47 ± 2.70	0.76

**Disease-specific clinical characteristics**			
Modified Hoehn and Yahr (n, %)			
Stage 1	3 (23.1)	1 (10.0)	
Stage 2	4 (30.8)	5 (50.0)	
Stage 2.5	5 (38.5)	4 (40.0)	0.97
Stage 3	1 (7.7)	--	

MDS- UPDRS I	8.53 ± 7.26	7.20 ± 6.23	0.51

MDS- UPDRS II	7.77 ± 5.73	7.50 ± 3.14	0.78

MDS- UPDRS III	26.61 ± 10.47	27.40 ± 5.60	0.56

Disease duration (years)	4.63 ± 2.85	4.70 ± 3.36	0.71

Core muscle endurance			

Prone bridge (s)	28.44 ± 13.27	33.48 ± 27.09	0.93

Sit-ups (repetitions/30 s)	17.61 ± 4.61	14.90 ±4.75	0.19

**Abdominal muscle thickness in supine position**			

TrA-Rest (cm)	0.34 ± 0.28	0.36 ± 0.10	0.54

IO-Rest (cm)	0.68 ± 0.18	0.67 ± 0.19	0.80

TrA-ADIM (cm)	0.54 ± 0.12	0.56 ± 0.15	0.62

IO-ADIM (cm)	0.82 ± 0.18	0.81 ± 0.27	0.76

**Abdominal muscle thickness in standing position**			

TrA-Rest (cm)	0.38 ± 0.11	0.28 ± 0.08	0.017*

IO-Rest (cm)	0.65 ± 0.19	0.52 ± 0.20	0.20

TrA-ADIM (cm)	0.56 ± 0.12	0.50 ± 0.23	0.13

IO-ADIM (cm)	0.88 ± 0.27	0.74 ± 0.36	0.28

SD: standard deviation, BMI: body mass index, MDS- UPDRS: movement disorder society-unified Parkinson’s disease rating scale, TrA: transversus abdominis, IO: internal obliques, ADIM: abdominal drawing-in maneuver,

* p < 0.05 Mann-Whitney U test.

**Table 3:** Comparison of the differences observed in muscle endurance and muscle thickness between assessments (pretraining and posttraining) in two patient groups.

Variable	Pilates group (n = 13) Δ Mean ± SD	Control group (n = 10) Δ Mean ± SD	p
**Core muscle endurance**			
Prone bridge (s)	18.02 ± 16.78	−1.27 ± 6.60	0.005*
Sit-ups (repetitions/30 s)	3.00 ± 3.36	−1.70 ± 2.31	0.002*
**Abdominal muscle thickness on supine position**			
TrA-Rest (cm)	0.03 ± 0.03	0.009 ± 0.024	0.710
IO-Rest (cm)	0.03 ± 0.09	−0.05 ± 0.05	0.008*
TrA-ADIM (cm)	0.07 ± 0.04	0.03 ± 0.09	0.063
IO-ADIM (cm)	0.05 ± 0.14	−0.08 ± 0.11	0.055
**Abdominal muscle thickness on standing position**			
TrA-Rest (cm)	0.03 ± 0.05	0.007 ± 0.03	0.100
IO-Rest (cm)	0.06 ± 0.12	−0.02 ± 0.08	0.067
TrA-ADIM (cm)	0.08 ± 0.06	0.072 ± 0.29	0.003*
IO-ADIM (cm)	0.13 ± 0.14	−0.05 ± 0.15	0.005*

SD: standard deviation, TrA: transversus abdominis, IO: internal obliques, ADIM: abdominal drawing-in maneuver,

* p < 0.05 Mann-Whitney U test.

**Table 4:** A comparison of previous and subsequent measurement of parameters for Pilates group.

Variable (n = 12)	Pretraining	Posttraining	Follow-up	p
**Core muscle endurance**				
Prone bridge (s)	28.04 ± 13.78^a^,^b^	47.26 ± 23.74	53.53±36.92	0.002*
Sit-ups (repetitions/30 s)	18.25 ± 4.18^a^	21.5 ± 4.58	19.5 ± 4.68	0.008*
**Abdominal muscle thickness in supine position**				
TrA-Rest (cm)	0.33 ± 0.06^b^	0.36 ± 0.06	0.35 ± 0.06	0.007*
IO-Rest (cm)	0.66 ± 0.18	0.69 ± 0.15	0.67 ± 0.17	0.472
TrA-ADIM (cm)	0.52 ± 0.12^a^,^b^	0.60 ± 0.12	0.58 ± 0.12	0.013*
IO-ADIM (cm)	0.81 ± 0.18	0.85 ± 0.18	0.85 ± 0.15	0.717
**Abdominal muscle thickness in standing position**				
TrA-Rest (cm)	0.37 ± 0.10	0.41 ± 0.11	0.40 ± 0.09	0.247
IO-Rest (cm)	0.63 ± 0.19	0.71 ± 0.18	0.68 ± 0.17	0.112
TrA-ADIM (cm)	0.54 ± 0.11^a^,^b^	0.63 ± 0.12	0.61 ± 0.13	0.000*
IO-ADIM (cm)	0.85 ± 0.27^a^	1.00 ± 0.19	0.99 ± 0.20	0.017*

SD: standard deviation, TrA: transversus abdominis, IO: internal obliques, ADIM: abdominal drawing-in maneuver,

* p < 0.05 Friedman test.

a Pretraining versus post-training p < 0.017.

b Pretraining versus follow-up p < 0.017.

a–b (Posthoc Wilcoxon signed ranks test with Bonferroni correction resulting in a significance level of p < 0.017.)

## References

[1] Tambosco L, Percebois-Macadré L, Rapin A, Nicomette-Bardel J, Boyer FC (2014). Effort training in Parkinson’s disease: a systematic review. Annals of Physical and Rehabilitation Medicine.

[2] Tomlinson CL, Patel S, Meek C, Herd C, Clarke C (2013). Physiotherapy versus placebo or no intervention in Parkinson’s disease. Cochrane Database of Systematic Reviews (CDSR).

[3] Clarke CE, Patel S, Ives N, Rick EC, Woolley R (2016). Clinical effectiveness and cost-effectiveness of physiotherapy and occupational therapy versus no therapy in mild to moderate Parkinson’s disease: a large pragmatic randomised controlled trial (PD REHAB). Health Technology Assessment.

[4] de Oliveira Francisco C, de Almeida Fagundes A, Gorges B (2015). Effects of Pilates method in elderly people: Systematic review of randomized controlled trials. Journal of Bodywork and Movement Therapies.

[5] de Freitas MLM, Zager M, Camphell C (2015). The influence of Pilates method on the postural instability of elderly patient with Parkinson disease. Fisioterapia Brasil.

[6] Mollinedo-Cardalda I, Carral JMC, Rodriguez-Fuentes G (2016). Pilates method guidelines for physical therapy in patients with Parkinson’s disease. Parkinsonism & Related Disorders.

[7] Johnson L, Putrino D, James I, Rodrigues J, Stell R (2013). The effects of a supervised Pilates training program on balance in Parkinson’s disease. Advances in Parkinson’s Disease.

[8] Muscolino JE, Cipriani S (2004). Pilates and the “powerhouse”-I. Journal of Bodywork and Movement Therapies.

[9] García-Jaén M, Cortell-Tormo JM, Hernández-Sánchez S, Tortosa-Martinez J (2020). Influence of abdominal hollowing maneuver on the core musculature activation during the prone plank exercise. International Journal of Environmental Research and Public Health.

[10] Atılgan E, Karaduman A (2016). Fizyoterapide Pilates. Fizyoterapi Rehabilitasyon.

[11] Critchley DJ, Pierson Z, Battersby G (2011). Effect of pilates mat exercises and conventional exercise programmes on transversus abdominis and obliquus internus abdominis activity: pilot randomised trial. Manuel Therapy.

[12] Suruliraj K, Chakrapani M, Ganesan S, Ellajosyla R (2017). Pelvic alignment in standing, and its relationship with trunk control and motor recovery of lower limb after stroke. Neurology and Clinical Neuroscience.

[13] Cole MH, Naughton GA, Silburn PA (2017). Neuromuscular impairments are associated with impaired head and trunk stability during gait in Parkinson fallers. Neurorehabilitation and Neural Repair.

[14] Masaki M, Kasahara M, Takeuchi M, Minakawa K, Inagaki Y (2022). Comparison of the mass and amount of intramuscular non-contractile tissue of the trunk and lower extremity muscles between patients with Parkinson’s disease and community-dwelling older adults. Neurological Sciences.

[15] Ota M, Ikezoe T, Kato T, Tateuchi H, Ichihashi N (2020). Age related changes in muscle thickness and echo intensity of trunk muscles in healthy women: comparison of 20–60s age groups. European Journal of Applied Physiology.

[16] Masaki M, Kasahara M, Inagaki Y, Yokota M, Takeuchi M (2023). Association of sagittal spinal alignment in the standing position with the masses and amounts of intramuscular non-contractile tissue of the trunk and lower extremity muscles in patients with Parkinson’s disease. Clinical Biomechanics.

[17] Moon JH, Hong SM, Kim CW, Shin Y (2015). Comparison of deep and superficial abdominal muscle activity between experienced Pilates and resistance exercise instructors and controls during stabilization exercise. Journal of Exercise Rehabilitation.

[18] Hebert JJ, Koppenhaver SL, Parent EC, Fritz JM (2009). A systematic review of the reliability of rehabilitative ultrasound imaging for the quantitative assessment of the abdominal and lumbar trunk muscles. Spine.

[19] Koppenhaver SL, Hebert JJ, Parent EC, Fritz JM (2009). Rehabilitative ultrasound imaging is a valid measure of trunk muscle size and activation during most isometric sub-maximal contractions: a systematic review. Australian Journal of Physiotherapy.

[20] Taghipour M, Mohseni-Bandpei MA, Behtash H, Abdollahi A, Rajabzadeh F (2019). Reliability of real-time ultrasound imaging for the assessment of trunk stabilizer muscles: A systematic review of the literature. Journal of Ultrasound in Medicine.

[21] Arab AM, Chehrehrazi M (2011). Ultrasound measurement of abdominal muscles activity during abdominal hollowing and bracing in women with and without stress urinary incontinence. Manuel Therapy.

[22] Park SY, Oh S, Baek KH, Bae SS, Kwon JW (2022). Comparison of abdominal muscle thickness between the abdominal draw-in maneuver and maximum abdominal contraction maneuver. Healthcare.

[23] Ko YH, Ha YG, Juri J, Van Hee L (2014). Variations in lateral abdominal muscle thickness during abdominal drawing-in maneuver in three positions in a young healthy population. Physical therapy rehabilitation science.

[24] Mew R (2009). Comparison of changes in abdominal muscle thickness between standing and crook lying during active abdominal hollowing using ultrasound imaging. Manual Therapy.

[25] Giacomini MB, da Silva AMV, Weber LM, Monterio MB (2016). The Pilates Method increases respiratory muscle strength and performance as well as abdominal muscle thickness. Journal of Bodywork and Movement Therapies.

[26] Han JS, Cho WS, Lim JH (2017). The effects of pilates mat exercise on trunk muscle thickness and balance. The Journal of Korean Physical Therapy.

[27] Öksüz S, Ünal E (2017). The effect of the clinical pilates exercises on kinesiophobia and other symptoms related to osteoporosis: Randomised controlled trial. Complementary Therapies in Clinical Practice.

[28] Goetz CG, Poewe W, Rascol O, Sampaio C, Stebbins GT (2004). Movement Disorder Society Task Force report on the Hoehn and Yahr staging scale: status and recommendations the Movement Disorder Society Task Force on rating scales for Parkinson’s disease. Movement Disorders.

[29] Goetz CG, Tilley BC, Shaftman SR, Stebbins GT, Fahn S (2008). Movement Disorder Society-sponsored revision of the Unified Parkinson’s Disease Rating Scale (MDS-UPDRS): scale presentation and clinimetric testing results. Movement Disorders.

[30] Akbostanci MC, Bayram E, Yilmaz V, Rzayev S, Özkan S (2018). Turkish standardization of movement disorders society unified Parkinson’s disease rating scale and unified dyskinesia rating scale. Movement Disorders Clinical Practice.

[31] Linek P, Saulicz E, Wolny T, Mysliwiec A (2017). Assessment of the abdominal muscles at rest and during abdominal drawing-in manoeuvre in adolescent physically active girls: A case–control study. Journal of Sport and Health Science.

[32] Tahan N, Khademi-Kalantari K, Mohseni-Bandpei MA, Mikaili S, Baghban AA (2016). Measurement of superficial and deep abdominal muscle thickness: an ultrasonography study. Journal of Physiological Anthropology.

[33] Whittaker JL, Stokes M (2011). Ultrasound imaging and muscle function. Journal of Orthopaedic & Sports Physical Therapy.

[34] Bulguroglu I, Guclu-Gunduz A, Yazici G, Özkul C, Irkec C (2017). The effects of Mat Pilates and Reformer Pilates in patients with Multiple Sclerosis: A randomized controlled study. NeuroRehabilitation.

[35] Dorado C, Calbet J, Lopez-Gordillo A, Alayon S, Sanchis-Moysi J (2012). Marked effects of Pilates on the abdominal muscles: a longitudinal magnetic resonance imaging study. Medicine & Science in Sports & Exercise.

[36] Çelenay ŞT, Kaya DÖ (2017). An 8-week thoracic spine stabilization exercise program improves postural back pain, spine alignment, postural sway, and core endurance in university students: a randomized controlled study. Turkish Journal of Medical Sciences.

[37] Jamali Brayjani S, Rahnama N, Abrishamkar S (2019). The Effect of Pilates Exercises on Muscular Endurance of the Central Part of Body and the Range of Motion of Lumbar Spine in Patients with Spondylolysis. Journal of paramedical sciences and rehabilitation.

[38] Llewellyn H, Konstantaki M, Johnson M, Francis P (2017). The effect of a Pilates exercise programme on perceived functional disability and pain associated with non-specific chronic low back pain. MOJ Yoga Physical Therapy.

[39] Monjo H, Fukumoto Y, Asai T, Shuntoh H (2018). Muscle thickness and echo intensity of the abdominal and lower extremity muscles in stroke survivors. Journal of Clinical Neurology.

[40] Lee J, Jeongwoob J, Dongyeopb L, Jiheonb H (2020). Effect of trunk stabilization exercise on abdominal muscle thickness, balance and gait abilities of patients with hemiplegic stroke: A randomized controlled trial. NeuroRehabilitation.

[41] Kim Y, Kim J, Nam H, Kim HY, Eom M (2020). Ultrasound imaging of the trunk muscles in acute stroke patients and relations with balance scales. Annals of Rehabilitation Medicine.

[42] Madokoro S, Yokogawa M, Miaki H (2020). Effect of the abdominal draw-in maneuver and bracing on abdominal muscle thickness and the associated subjective difficulty in healthy individuals. Healthcare.

